# Dynamics of a Delayed Model for the Transmission of Malicious Objects in Computer Network

**DOI:** 10.1155/2014/194104

**Published:** 2014-07-23

**Authors:** Zizhen Zhang, Huizhong Yang

**Affiliations:** ^1^School of Management Science and Engineering, Anhui University of Finance and Economics, Bengbu 233030, China; ^2^Key Laboratory of Advanced Process Control for Light Industry (Ministry of Education), Jiangnan University, Wuxi 214122, China

## Abstract

An SEIQRS model for the transmission of malicious objects in computer network with two delays is investigated in this paper. We show that possible combination of the two delays can affect the stability of the model and make the model bifurcate periodic solutions under some certain conditions. For further investigation, properties of the periodic solutions are studied by using the normal form method and center manifold theory. Finally, some numerical simulations are given to justify the theoretical results.

## 1. Introduction

Computer viruses in network have posed a major threat to our work and life with the rapid popularization of the Internet. Many virus propagation models [1–4] have been proposed to understand the way that computer viruses propagate after Kephart and White [5] proposed the first epidemiological model of computer viruses. In [1], Thommes and Coates proposed a modified version of the SEI model to predict the virus propagation in a network. In [3], Wen and Zhong studied an SIR model on bipartite networks and they proved the existence and the asymptotic stability of the endemic equilibrium by applying the theory of the multigroup model. In [4], Mishra and Jha proposed the following SEIQRS model to describe the transmission of malicious objects in computer network by introducing a new compartment quarantine into the SEIRS model proposed in [2]:

(1) dS(t)dt=A−βS(t)I(t)−dS(t)+ηR(t),dE(t)dt=βS(t)I(t)−(d+μ)E(t),dI(t)dt=μE(t)−(d+α+γ+δ)I(t),dQ(t)dt=δI(t)−(d+α+ɛ)Q(t),dR(t)dt=γI(t)+ɛQ(t)−(d+η)R(t),

where *S*(*t*), *E*(*t*), *I*(*t*), *Q*(*t*), and *R*(*t*) denote the sizes of nodes in the states susceptible, exposed, infectious, quarantined, and recovered at time *t*, respectively. *A* is the rate at which new computers are attached to the network. *d* is the rate at which computers are disconnected to the network. *α* is the crashing rate of computers due to the attack of malicious objects. *β* is the transmission rate. *μ*, *γ*, *δ*, *ɛ*, and *η* are the state transition rates.

As is known, an infected computer becomes a recovered one by using antimalicious software and the recovered computer has a temporary immunity, and computer virus models with delay have been studied by many scholars [6–12]. In [6], Ren et al. investigated local and global stability of a delayed viral infection model in computer virus propagation model. In [8], Dong et al. proposed a delayed SEIR computer virus model and studied the problem of Hopf bifurcation of the model by regarding the delay as a bifurcating parameter. Motivated by the work above, Liu [12] incorporated the time delay due to the temporary immunity period into system (1) and proposed the following SEIQRS model with time delay:

(2) dS(t)dt=A−βS(t)I(t)−dS(t)+ηR(t−τ),dE(t)dt=βS(t)I(t)−(d+μ)E(t),dI(t)dt=μE(t)−(d+α+γ+δ)I(t),dQ(t)dt=δI(t)−(d+α+ɛ)Q(t),dR(t)dt=γI(t)+ɛQ(t)−dR(t)−ηR(t−τ),

where *τ* ≥ 0 is the time delay due to the temporary immunity period. However, we know that an infected computer needs a period to clean viruses by antivirus software and then becomes a recovered one. Therefore, there is a time delay before the infected computers develop themselves into the recovered ones. And there have been some papers that deal with the research of Hopf bifurcation of dynamical system with multiple delays [13–18]. In [13], Xu and He considered a two-neuron network with resonant bilinear terms and two delays. They studied the problem of Hopf bifurcation by regarding the sum of the two delays as a bifurcation parameter. In [16], Meng et al. studied the Hopf bifurcation of a three-species system with two delays by regarding possible combination of the two delays as a bifurcation parameter. Motivated by the work above, we consider the following SEIQRS computer virus model with two delays in the present paper:

(3) dS(t)dt=A−βS(t)I(t)−dS(t)+ηR(t−τ1),dE(t)dt=βS(t)I(t)−(d+μ)E(t),dI(t)dt=μE(t)−(d+α+δ)I(t)−γI(t−τ2),dQ(t)dt=δI(t)−(d+α)Q(t)−ɛQ(t−τ2),dR(t)dt=γI(t−τ2)+ɛQ(t−τ2)−dR(t)−ηR(t−τ1),

where *τ*_1_ is the time delay due to the temporary immunity period and *τ*_2_ is the time delay due to the period that the infected computer uses to clean viruses by antivirus software.

The main purpose of this paper is to investigate the effects of the two delays on system (3) and the remainder of this paper is organized as follows. Sufficient conditions for local stability and existence of local Hopf bifurcation are obtained by analyzing the distribution of the roots of the associated characteristic equation in Section 2. Properties of the Hopf bifurcation are further investigated by using the normal form method and center manifold theory in Section 3. In Section 4, we give a numerical example to support the theoretical results in the paper.

## 2. Local Stability and Existence of Local Hopf Bifurcation

By a simple computation, it is easy to get that if *R*_0_ = *Aμβ*/*d*(*d* + *μ*)(*α* + *δ* + *γ* + *d*) > 1, then system (3) has a unique positive equilibrium P*(S*, E*, I*, Q*, R*), where

(4) $$\begin{array}{c} S^{*}=\frac{\left(d+\mu \right)\left(d+\alpha +\delta +\gamma \right)}{\mu \beta },\;\;E^{*}=\frac{\left(d+\alpha +\delta +\gamma \right)I^{*}}{\mu },\\ I^{*}=\frac{\left(d+\eta \right)\left(d+\alpha +ɛ\right)\left(A\mu \beta -d\left(d+\mu \right)\left(d+\alpha +\delta +\gamma \right)\right)}{\beta },\\ Q^{*}=\frac{\delta I^{*}}{d+\alpha +ɛ},\;\;R^{*}=\frac{\gamma I^{*}}{d+\eta }+\frac{ɛ\delta I^{*}}{\left(d+\eta \right)\left(d+\alpha +ɛ\right)}, \end{array}$$

and *R*_0_ is the basic reproduction number. It is easy to get the linearization of system (3) at P*(S*, E*, I*, Q*, R*):

(5) dS(t)dt=a11S(t)+a13I(t)+b15R(t−τ1),dE(t)dt=a21S(t)+a22E(t)+a23I(t),dI(t)dt=a32E(t)+a33I(t)+c33I(t−τ2),dQ(t)dt=a43I(t)+a44Q(t)+c44Q(t−τ2),dR(t)dt=a55R(t)+b55R(t−τ1)+c53I(t−τ2)+c54Q(t−τ2),

where

(6) $$\begin{array}{c} a_{11}=-\left(\beta I^{*}+d\right),\;\;a_{13}=-\beta S^{*},\;\;a_{21}=\beta I^{*},\\ a_{22}=-\left(d+\mu \right),\;\;a_{23}=\beta S^{*},\;\;a_{32}=\mu ,\\ a_{33}=-\left(d+\alpha +\delta \right),\;\;a_{43}=\delta ,\;\;a_{44}=-\left(d+\alpha \right),\\ a_{55}=-d,\;\;b_{15}=\eta ,\;\;b_{55}=-\eta ,\;\;c_{33}=-\gamma ,\\ c_{44}=-ɛ,\;\;c_{53}=\gamma ,\;\;c_{54}=ɛ. \end{array}$$

Thus, the characteristic equation of system (5) is

(7) λ5+m4λ4+m3λ3+m2λ2+m1λ+m0+(n4λ4+n3λ3+n2λ2+n1λ+n0)e−λτ1+(p4λ4+p3λ3+p2λ2+p1λ+p0)e−λτ2+(q3λ3+q2λ2+q1λ+q0)e−2λτ2+(r3λ3+r2λ2+r1λ+r0)e−λ(τ1+τ2)+(s2λ2+s1λ+s0)e−λ(τ1+2τ2)=0,

where

(8) m0=(a11a23a32−a11a22a33−a13a21a32)a44a55,m1=(a44+a55)(a13a21a32−a11a23a32)−a23a32a44a55   +a11a22a33a44+a11a22a55(a33+a44)   +a33a44a55(a11+a22),m2=a23a32(a11+a44+a55)−a55(a11a22+a33a44)   −a13a21a32−a11a22(a33+a44)−a33a44(a11+a22)   −a55(a11+a22)(a33+a44),m3=a11a22+a33a44+(a11+a22)(a33+a44)−a23a32   +a55(a11+a22+a33+a44),m4=−(a11+a22+a33+a44+a55),n0=(a11a23a32−a11a22a33−a13a21a32)a44b55,n1=a11a22b55(a33+a44)+a33a44b55(a11+a22)  +a13a21a32b55−a23a32b55(a11+a44),n2=(a23a32−a11a22−a33a44)b55  −(a11+a22)(a33+a44)b55,n3=b55(a11+a22+a33+a44),  n4=−b55,p0=(a11a23−a13a21)a32a55c44−(a44c33+a33c44)a11a22a55,p1=a11a22c33(a44+a55)+a44a55c33(a11+a22)  +a11a22c44(a33+a55)−a23a32c44(a11+a55)  +a33a55c44(a11+a22)−a13a21a32c44,p2=a23a32c44−c33(a11+a22)(a44+a55)  −c33(a11a22+a44a55)−c44(a11+a22)(a33+a55)  −c44(a11a22+a33a55),p3=c33(a11+a22+a44+a55)+c44(a11+a22+a33+a55),p4=−(c33+c44),  q0=a11a22a55c33c44,  q3=c33c44,q1=(a11a22+a11a55+a22a55)c33c44,q2=−(a11+a22+a55)c33c44,r0=a21a32b15(a44c53−a43c54)−a11a22b55(a33c44+a44c33)  −a32b55c44(a11a23+a13a21),r1=(a11a22+a11a44+a22a44)b55c33−a21c32b15c53  +(a11a22+a11a33+a22a33)b55c44+a23a32b55c44,r2=−(a11+a22+a33)b55c44−(a11+a22+a44)b55c33,r3=(c33+c44)b55,  s0=a21a32b15c44c53−a11a22b55c33c44,s1=(a11+a22)b55c33c44,  s2=−b55c33c44.

**Case 1 (*τ*_1_ = *τ*_2_ = 0).**

When *τ*_1_ = *τ*_2_ = 0, (7) becomes

(9) λ5+A14λ4+A13λ3+A12λ2+A11λ+A10=0,A10=m0+n0+p0+q0+r0+s0,A11=m1+n1+p1+q1+r1+s1,A12=m2+n2+p2+q2+r2+s2,A13=m3+n3+p3+q3+r3,A14=m4+n4+p4=βI∗+5d+2α+μ+δ+ɛ+γ+η>0.

Let det_1_ = *A*_14_. Obviously, det_1_ > 0. Therefore, if the condition (*H*_1_): (10) holds, then the positive equilibrium P*(S*, E*, I*, Q*, R*) of system (3) is locally asymptotically stable without delay. Consider

(10) det2=|A141A12A13|>0,det3=|A1410A12A13A140A11A12|>0,det4=|A14100A12A13A141A10A11A12A1300A10A11|>0,det5=|A141000A12A13A1410A10A11A12A13A1400A10A11A120000A10|>0.

**Case 2 (*τ*_1_ > 0, *τ*_2_ = 0).**

When *τ*_2_ = 0, (7) becomes the following form:

(11) λ5+A24λ4+A23λ3+A22λ2+A21λ+A20 +(B24λ4+B23λ3+B22λ2+B21λ+B20)e−λτ1=0,

where

(12) $$\begin{array}{c} A_{20}=m_{0}+p_{0}+q_{0},\;\;A_{21}=m_{1}+p_{1}+q_{1},\\ A_{22}=m_{2}+p_{2}+q_{2},\;\;A_{23}=m_{3}+p_{3}+q_{3},\\ A_{24}=m_{4}+p_{4},\;\;B_{20}=n_{0}+r_{0}+s_{0},\\ B_{21}=n_{1}+r_{1}+s_{1},\;\;B_{22}=n_{2}+r_{2}+s_{2},\\ B_{23}=n_{3}+r_{3},\;\;B_{24}=n_{4}. \end{array}$$

Let *λ* = *iω*_1_ (*ω*_1_ > 0) be a root of (11). Then, we obtain

(13) (B21ω1−B23ω13)sinτ1ω1+(B24ω14−B22ω12+B20)cos⁡τ1ω1 =A22ω12−A24ω14−A20,(B21ω1−B23ω13)cos⁡τ1ω1−(B24ω14−B22ω12+B20)sinτ1ω1 =−ω15+A23ω13−A21ω1.

It follows that

(14) $$\begin{array}{c} \omega_{1}^{10}+c_{24}\omega_{1}^{8}+c_{23}\omega_{1}^{6}+c_{22}\omega_{1}^{4}+c_{21}\omega_{1}^{2}+c_{20}=0, \end{array}$$

with

(15) c20=A202−B202,c21=A212−B212−2A20A22+2B20B22,c22=A222−B222+2A20A24−2A21A23−2B20B24+2B21B23,c23=A232+2A21−2A22A24−B232+2B22B24,c24=A242−B242−2A23.

Let *ω*_1_^2^ = *v*_1_, then (14) becomes

(16) $$\begin{array}{c} v_{1}^{5}+c_{24}v_{1}^{4}+c_{23}v_{1}^{3}+c_{22}v_{1}^{2}+c_{21}v_{1}+c_{20}=0. \end{array}$$

If all the parameters of system (3) are given, one can get all the roots of (16) by the software package Matlab. In order to give the main results in this paper, we make the following assumption.

(*H*_21_)  (16) has at least one positive real root.

If the condition (*H*_21_) holds, then there exists a *v*_10_ such that (11) has a pair of purely imaginary roots $\pm i\omega_{10}=\pm i\sqrt{v_{10}}$. For *ω*_10_, the corresponding critical value of time delay is

(17) $$\begin{array}{c} \tau_{10}=\frac{1}{\omega_{10}}\text{arccos}\frac{p_{28}\omega_{10}^{8}+p_{26}\omega_{10}^{6}+p_{24}\omega_{10}^{4}+p_{22}\omega_{10}^{2}+p_{20}}{q_{28}\omega_{10}^{8}+q_{26}\omega_{10}^{6}+q_{24}\omega_{10}^{4}+q_{22}\omega_{10}^{2}+q_{20}}, \end{array}$$

where

(18) p20=−A20B20,  p22=A20B22−A21B21+A22B20,p24=A21B23+A23B21−A20B24−A22B22−A24B20,p26=A22B24+A24B22−A23B23−B21,p28=B23−A24B24,  q20=B202,q22=B212−2B20B22,  q24=B222+2B20B24−2B21B23,q26=B232−2B22B24,  q28=B242.

Taking the derivative of *λ* with respect to *τ*_1_ in (11), one can obtain

(19) [dλdτ1]−1=−5λ4+4A24λ3+3A23λ2+2A22λ+A21λ(λ5+A24λ4+A23λ3+A22λ2+A21λ+A20) +4B24λ3+3B23λ2+2B22λ+B21λ(B24λ4+B23λ3+B22λ2+B21λ+B20)−τ1λ.

Thus,

(20) Re[dλdτ1]λ=iω10−1 =f1′(v1∗)(B21ω10−B23ω103)2+(B24ω104−B22ω102+B20)2,

where *f*_1_(*v*_1_) = *v*_1_^5^ + *c*_24_ *v*_1_^4^ + *c*_23_ *v*_1_^3^ + *c*_22_ *v*_1_^2^ + *c*_21_ *v*_1_ + *c*_20_ and *v*_1∗_ = *ω*_10_^2^.

Obviously, if the condition (*H*_22_)  *f*_1_′(*v*_1∗_) ≠ 0 holds, then *Re*[*dλ*/*dτ*_1_]_*λ*=*iω*_10__^−1^ ≠ 0. According to the Hopf bifurcation theorem in [19], we have the following results for system (3).

**Theorem 1 .**

For system (3), if the conditions (*H*_21_)-(*H*_22_) hold, then the positive equilibrium P*(S*, E*, I*, Q*, R*) of system (3) is asymptotically stable for *τ*_1_ ∈ [0, *τ*_10_) and system (3) undergoes a Hopf bifurcation at the positive equilibrium P*(S*, E*, I*, Q*, R*) when *τ*_1_ = *τ*_10_.

**Case 3 (*τ*_1_ = 0, *τ*_2_ > 0).**

When *τ*_1_ = 0, (7) becomes

(21) λ5+A34λ4+A33λ3+A32λ2+A31λ+A30+(B34λ4+B33λ3+B32λ2+B31λ+B20)e−λτ2+(C33λ3+C32λ2+C30)e−2λτ2=0,

where

(22) $$\begin{array}{c} A_{30}=m_{0}+n_{0},\;\;A_{31}=m_{1}+n_{1},\;\;A_{32}=m_{2}+n_{2},\\ A_{33}=m_{3}+n_{3},\;\;A_{34}=m_{4}+n_{4},\;\;B_{30}=p_{0}+r_{0},\\ B_{31}=p_{1}+r_{1},\;\;B_{32}=p_{2}+r_{2},\;\;B_{33}=p_{3}+r_{3},\\ B_{34}=p_{4},\;\;C_{30}=q_{0}+s_{0},\;\;C_{31}=q_{1}+s_{1},\\ C_{32}=q_{2}+s_{2},\;\;C_{33}=q_{3}. \end{array}$$

Multiplying *e* ^*λτ*_2_^ on both sides of (21), we have

(23) B34λ4+B33λ3+B32λ2+B31λ+B20 +(λ5+A34λ4+A33λ3+A32λ2+A31λ+A30)eλτ2 +(C33λ3+C32λ2+C30)e−λτ2=0.

Let *λ* = *iω*_2_ (*ω*_2_ > 0) be the root of (23), then we obtain

(24) $$\begin{array}{c} M_{31}\sin\tau_{2}\omega_{2}+M_{32}\cos\tau_{2}\omega_{2}=M_{33},\\ M_{34}\cos\tau_{2}\omega_{2}-M_{35}\sin\tau_{2}\omega_{2}=M_{36}, \end{array}$$

where

(25) M31=A34ω24+(C32−A32)ω22+A30−C30,M32=ω25−(A33+C33)ω23+(A31+C31)ω2,M33=B33ω23−B31ω2,M34=A34ω24−(A32+C32)ω22+A30+C30,M35=ω25−(A33−C33)ω23+(A31−C31)ω2,M36=B32ω22−B34ω24−B30.

Then, we obtain

(26) $$\begin{array}{c} \cos\tau_{2}\omega_{2}=\frac{p_{38}\omega_{2}^{8}+p_{36}\omega_{2}^{6}+p_{34}\omega_{2}^{4}+p_{32}\omega_{2}^{2}+p_{30}}{\omega_{2}^{10}+q_{38}\omega_{2}^{8}+q_{36}\omega_{2}^{6}+q_{34}\omega_{2}^{4}+q_{32}\omega_{2}^{2}+q_{30}},\\ \sin\tau_{2}\omega_{2}=\frac{p_{37}\omega_{2}^{7}+p_{35}\omega_{2}^{5}+p_{33}\omega_{2}^{3}+p_{31}\omega_{2}}{\omega_{2}^{10}+q_{38}\omega_{2}^{8}+q_{36}\omega_{2}^{6}+q_{34}\omega_{2}^{4}+q_{32}\omega_{2}^{2}+q_{30}}, \end{array}$$

where

(27) p30=A30(C30−B30),p31=(A31+C31)B30−(A30+C30)B31,p32=B31(C31−A31)+A32B30+A30B32−B30C32   −B32C30,p33=B31(A32+C32)+B33(A30+C30)−B30(A33+C33)   −B32(A31+C31),p34=B31(A33−C33)+B32(C32−A32)+B33(A31−C31)   +B34(C30−A30)−A34B30,p35=B32(A33+C33)+B34(A31+C31)−B33(A32+C32)   −A34B31,p36=A34B32+A32B34−A33B33−B31+B33C33−B34C32,p37=A34B33−B32−(A33+C33)B34,p38=B33−A34C34,q30=A302−C302,  q32=A312−C312−2A30A32+2C30C32,q34=A322−C322−2A30A34+2C31C33−2A31A33,q36=A332−C332+2A31−2A32A34,  q38=A342−2A33.

Then, we obtain

(28) ω220+c39ω218+c38ω216+c37ω214+c36ω212+c35ω210+c34ω28+c33ω26+c32ω24+c31ω22+c30=0,

where

(29) c30=q302−p302,  c31=2q30q32−2p30p32−p312,c32=q322−p322+2q30q34−2p30p34−2p31p33,c33=2q30q36+2q32q34−2p30p36−2p31p35   −2p32p34−p332,c34=q342−p342+2q30q38+2q32q36−2p30p38   −2p31p37−2p32p36−2p33p35,c35=2q32q38−2p32p38−2p33p37−2p34p36−p352,c36=q362−p362+2q34q38+2q32−2p34p38−2p35p37,c37=2q34+2q36q38−p36p38−p372,c38=q382−p382+2q36,  c39=2q38.

Let *ω*_2_^2^ = *v*_2_, then (28) becomes

(30) v210+c39v29+c38v28+c37v27+c36v26+c35v25+c34v24+c33v23+c32v22+c31v2+c30=0.

Similar as in Case 2, we make the following assumption. (*H*_31_) (30) has at least one positive real root. If the condition (*H*_31_) holds, then there exists a *v*_20_ such that (23) has a pair of purely imaginary roots $\pm i\omega_{20}=\pm i\sqrt{v_{20}}$. For *ω*_20_, the corresponding critical value of time delay is

(31) τ20=1ω20arccosp38ω208+p36ω206+p34ω204+p32ω202+p30ω2010+q38ω208+q36ω206+q34ω204+q32ω202+q30.

Differentiating two sides of (23) with respect to *τ*_2_, we have

(32) $$\begin{array}{c} {\left[\frac{d\lambda }{d\tau_{2}}\right]}^{-1}=\frac{g_{31}\left(\lambda \right)+g_{32}\left(\lambda \right)e^{\lambda \tau_{2}}+g_{33}\left(\lambda \right)e^{-\lambda \tau_{2}}}{g_{34}\left(\lambda \right)e^{-\lambda \tau_{2}}-g_{35}\left(\lambda \right)e^{\lambda \tau_{2}}}-\frac{\tau_{2}}{\lambda }, \end{array}$$

where

(33) g31(λ)=4B34λ3+3B33λ2+2B32λ+B31,g32(λ)=5λ4+4A34λ3+3A33λ2+2A32λ+A31,g33(λ)=3C33λ2+2C32λ+C31,g34(λ)=C33λ4+C32λ3+C31λ2+C30λ,g35(λ)=λ6+A34λ5+A33λ4+A32λ3+A31λ2+A30λ.

Thus,

(34) Re[dλdτ2]λ=iω20−1=P3RQ3R+P3IQ3IQ3R2+Q3I2,

where

(35) P3R=(5ω204−3(A33+C33)5ω202+A31+C31)cos⁡τ20ω20   +(4A34ω203+2(C32−A32)ω20)sinτ20ω20+B31   −3B33ω202,P3I=(5ω204−3(A33−C33)5ω202+A31−C31) sinτ20ω20   −(4A34ω203−2(A32+C32)ω20)cos⁡τ20ω20+2B32ω20   −4B34ω203,Q3R=((A33+C33)ω204−ω206−(A31+C31)ω202)cos⁡τ20ω20   −(A34ω205+(A32−C32)ω203+(A30−C30)ω20)   ×sinτ20ω20,Q3I=((A33−C33)ω204−ω206−(A31−C31)ω202)sinτ20ω20   +(A34ω205−(A32+C32)ω203+(A30+C30)ω20)   ×cos⁡τ20ω20.

Obviously, if the condition (*H*_32_)  *P*_3*R*_ *Q*_3*R*_ + *P*_3*I*_ *Q*_3*I*_ ≠ 0 holds, then *Re*[*dλ*/*dτ*_2_]_*λ*=*iω*_20__^−1^ ≠ 0. According to the Hopf bifurcation theorem in [19], we have the following results for system (3).

**Theorem 2 .**

For system (3), if the conditions (*H*_31_)-(*H*_32_) hold, then the positive equilibrium P*(S*, E*, I*, Q*, R*) of system (3) is asymptotically stable for *τ*_2_ ∈ [0, *τ*_20_) and system (3) undergoes a Hopf bifurcation at the positive equilibrium P*(S*, E*, I*, Q*, R*) when *τ*_2_ = *τ*_20_.

**Case 4 (*τ*_1_ > 0, *τ*_2_ > 0, *τ*_2_ ∈ (0, *τ*_20_)).**

We consider system (3) under the condition that *τ*_2_ is in its stable interval and *τ*_1_ is a bifurcation parameter.

Let *λ* = *iω*_1∗_ (*ω*_1∗_ > 0) be the root of (7), then we obtain

(36) $$\begin{array}{c} M_{41}\sin\tau_{1}\omega_{1*}+M_{42}\cos\tau_{1}\omega_{1*}=M_{43},\\ M_{41}\cos\tau_{1}\omega_{1*}-M_{42}\sin\tau_{1}\omega_{1*}=M_{44}, \end{array}$$

where

(37) M41=n1ω1∗−n3ω1∗3+(r1ω1∗−r3ω1∗3)cos⁡τ2ω1∗    −(r0−r2ω1∗2)sinτ2ω1∗+s1ω1∗cos⁡2τ2ω1∗    −(s0−s2ω1∗2)sin2τ2ω1∗,M42=n4ω1∗4−n2ω1∗2+n0+(r1ω1∗−r3ω1∗3)sinτ2ω1∗    +(r0−r2ω1∗2)cos⁡τ2ω1∗+s1ω1∗sin2τ2ω1∗    +(s0−s2ω1∗2)cos⁡2τ2ω1∗,M43=(p3ω1∗3−p1ω1∗)sinτ2ω1∗    −(p4ω1∗4−p2ω1∗2+p0)cos⁡τ2ω1∗    +(q3ω1∗3−q1ω1∗)sin2τ2ω1∗    +(q2ω1∗2−q0)cos⁡τ2ω1∗+m2ω1∗2−m4ω1∗4−m0,M44=(p3ω1∗3−p1ω1∗)cos⁡τ2ω1∗    +(p4ω1∗4−p2ω1∗2+p0)sinτ2ω1∗    +(q3ω1∗3−q1ω1∗)cos⁡2τ2ω1∗    −(q2ω1∗2−q0)sinτ2ω1∗+m3ω1∗3−ω1∗5−m1ω1∗.

Then, we can obtain

(38) f40(ω1∗)+2f41(ω1∗)cos⁡τ2ω1∗+2f42(ω1∗)sinτ2ω1∗  +2f43(ω1∗)cos⁡2τ2ω1∗+2f44(ω1∗)sin2τ2ω1∗  +2f45(ω1∗)cos⁡τ2ω1∗cos⁡2τ2ω1∗  +2f46(ω1∗)cos⁡τ2ω1∗sin2τ2ω1∗  +2f47(ω1∗)sinτ2ω1∗sin2τ2ω1∗  +2f48(ω1∗)sinτ2ω1∗cos⁡2τ2ω1∗=0,

where

(39) f40(ω1∗)=ω1∗10+(m42−n42+p42−2m3)ω1∗8      +(m32−n32+p32+q32−r32+2m1−2m2m4         + 2n2n4−2p2p4)ω1∗6      +(m22−n22+p22+q22−r22−s22+2m0m4         −2m1m3+2n0n4+2n1n3−2p1p3−2q1q3         + 2r1r3)ω1∗4      +(m12−n12+p12+q12−r12−s12−2m0m2        +2n0n2−2p0p2−2q0q2+2r0r2        + 2s0s2)ω1∗2+m02−n02+p02+q02−r02−s02,f41(ω1∗)=(m4p4−p3)ω1∗8      +(m3p3+p1−m2p4−m4p2−n3r3+n4r2)ω1∗6      +(m0p4−m1p3+m2p2−m3p1+m4p0+n1r3       − n2r2+n3r1−n4r0)ω1∗4      +(m1p1−m0p2−m2p0+n0r2−n1r1       + n2r0)ω1∗2+m0p0−n0r0,f42(ω1∗)=−p4ω1∗9+(m3p4−m4p3+n4r3+p2)ω1∗7      +(m2p3−m1p4−m3p2+m4p1−n2r3       + n3r2−n4r1−p0)ω1∗5      +(m1p2−m0p3−m2p1+m3p0+n0r3       − n1r2+n2r1−n3r0)ω1∗3      +(m0p1−m1p0−n0r1+n1r0)ω1∗,f43(ω1∗)=−q3ω1∗8+(m3q3−m4q2+q1)ω1∗6      +(m2q2−m1q3−m3q1+m4q0+n3s1)ω1∗4      +(m1q1−m0q2−m2q0−n1s1)ω1∗2,f44(ω1∗)=q2ω1∗7+(n3s2−m3q2−q0)ω1∗5      +(m1q2+m3q0−n1s2−n3s0)ω1∗3      +(n1s0−m1q0)ω1∗,f45(ω1∗)=(p3q3−p4q2)ω1∗6      +(p2q2+p4q0−p1q3−p3q1+r2s2−r3s1)ω1∗4      +(p1q1−p0q2−p2q0−r0s2+r1s1−r2s0)ω1∗2      +p0q0+r0s0,f46(ω1∗)=−p4q3ω1∗7+(p2q3−p3q2+p4q1+r3s2)ω1∗5      +(p1q2−p0q3−p2q1−p3q0−r1s2       + r2s1−r3s0)ω1∗3      +(p0q1−p1q0−r1s0−r0s1)ω1∗,f47(ω1∗)=(p3q3−p4q2)ω1∗6      +(p2q2−p1q3−p3q1−p4q0−r2s2+r3s1)ω1∗4      +(p1q1−p0q2−p2q0+r0s2−r1s1+r2s0)ω1∗2      +p0q0−r0s0,f48(ω1∗)=p4q3ω1∗7+(p3q2−p2q3−p4q1−r3s2)ω1∗5      +(p0q3−p1q2+p2q1−p3q0+r1s2       − r2s1−r3s0)ω1∗3      +(p1q0+p0q1+r0s1−r1s0)ω1∗.

In order to give the main results in this paper, we make the following assumption.

(*H*_41_) (38) has at least one positive real root. If the conditions (*H*_41_) hold, then there exists a *ω*_10_* such that (7) has a pair of purely imaginary roots ±*iω*_10_*. For *ω*_10_*, the corresponding critical value of time delay is

(40) $$\begin{array}{c} \tau_{10}^{*}=\frac{1}{\omega_{10}^{*}}\text{arccos}{\frac{M_{41}M_{44}+M_{42}M_{43}}{M_{41}^{2}+M_{42}^{2}}|}_{\omega_{1*}=\omega_{10}^{*}}. \end{array}$$

Differentiating two sides of (7) with respect to *τ*_1_, we have

(41) [dλdτ1]−1=(g41(λ)+g42(λ)e−λτ1+g43(λ)e−λτ2  +g44(λ)e−λ(τ1+τ2)+g45(λ)e−2λτ2  + g46(λ)e−λ(τ1+2τ2)) ×(g47(λ)e−λτ1+g48(λ)e−λ(τ1+τ2)    + g49(λ)e−λ(τ1+2τ2))−1 −τ1λ,

where

(42) g41(λ)=5λ4+4m4λ3+3m3λ2+2m2λ+m1,g42(λ)=4n4λ3+3n3λ2+2n2λ+n1,g43(λ)=−τ2p4λ4+(4p4−τ2p3)λ3+(3p3−τ2p2)λ2     +(2p2+τ2p1)λ+p1−τ2p0,g44(λ)=−τ2q33+(3q3−τ2q2)2+(2q2−τ2p1)λ     +q1−τ2q0,g45(λ)=−τ2s2λ2+(2s2−τ2s1)λ+s1−τ2s0,g46(λ)=3r3λ2+2r2λ+r1,g47(λ)=n4λ5+n3λ4+n2λ3+n1λ2+n0λ,g48(λ)=r3λ4+r2λ3+r1λ2+r0λ,g49(λ)=s2λ3+s1λ2+s0λ.

Thus,

(43) Re[dλdτ1]λ=iω10∗−1=P4RQ4R+P4IQ4IQ4R2+Q4I2,

where

(44) P4R=5(ω10∗)4−3m3(ω10∗)2+m1   +(2n2ω10∗−4n4(ω10∗)3+2r2ω10∗cos⁡τ2ω10∗    +(3r3(ω10∗)2−r1)sinτ2ω10∗    +(2s2−τ2s1)ω10∗cos⁡2τ2∗ω10∗    − (τ2s2(ω10∗)2+s1−τ2s0)sin2τ2ω10∗)sinτ10∗ω10∗   +(n1−3n3(ω10∗)2+2r2ω10∗sinτ2ω10∗    +(r1−3r3(ω10∗)2)cos⁡τ2ω10∗    +(2s2−τ2s1)ω10∗sin2τ2ω10∗    + (τ2s2(ω10∗)2+s1−τ2s0)cos⁡2τ2ω10∗)cos⁡τ2ω10∗   +((2p2−τ2p1)ω10∗−(4p4−τ2p3)(ω10∗)3)sinτ2ω10∗   +((τ2p2−3p3)(ω10∗)2−τ2p4(ω10∗)4+p1−τ2p0)   ×cos⁡τ2ω10∗   +(τ2q3(ω10∗)3+(2q2−τ2q1)ω10∗)sin2τ2ω10∗   +((τ2q2−3q3)(ω10∗)2+q1−τ2q0)cos⁡2τ2ω10∗,P4I=2m2ω10∗−4m4(ω10∗)3+2r2ω10∗cos⁡τ2ω10∗   +((3r3(ω10∗)2−r1)sinτ2ω10∗    +(2s2−τ2s1)ω10∗cos⁡2τ2ω10∗    − (τ2s2(ω10∗)2+s1−τ2s0)sin2τ2ω10∗)cos⁡τ2ω10∗   +(3n3(ω10∗)2−n1−2r2ω10∗sinτ2ω10∗    −(r1−3r3(ω10∗)2)cos⁡τ2ω10∗    −(2s2−τ2s1)ω10∗sin2τ2ω10∗    −(τ2s2(ω10∗)2+s1−τ2s0)cos⁡2τ2ω10∗)sinτ10∗ω10∗   +((2p2−τ2p1)ω10∗−(4p4−τ2p3)(ω10∗)3)cos⁡τ2ω10∗   −((τ2p2−3p3)(ω10∗)2−τ2p4(ω10∗)4+p1−τ2p0)   ×sinτ2ω10∗   +(τ2q3(ω10∗)3+(2q2−τ2q1)ω10∗)cos⁡2τ2ω10∗   −((τ2q0−3q3)(ω10∗)2+q1−τ2q0)sin2τ2ω10∗,

(45) Q4R=(n4(ω10∗)5−n2(ω10∗)3+n0ω10∗    +(r0ω10∗−r2(ω10∗)3)cos⁡τ2ω10∗    −(r3(ω10∗)4−r1(ω10∗)2)sinτ2ω10∗    +(s0ω10∗−s2(ω10∗)3)cos⁡τ2ω10∗    + s1(ω10∗)2sin2τ2ω10∗)sinτ10∗ω10∗   +(n3(ω10∗)4−n1(ω10∗)2+(r0ω10∗−r2(ω10∗)3)sinτ2ω10∗    +(r3(ω10∗)4−r1(ω10∗)2)cos⁡τ2ω10∗    +(s0ω10∗−s2(ω10∗)3)sin2τ2ω10∗    − s1(ω10∗)2cos⁡2τ2ω10∗)cos⁡τ10∗ω10∗,Q4I=(n4(ω10∗)5−n2(ω10∗)3+n0ω10∗    +(r0ω10∗−r2(ω10∗)3)cos⁡τ2ω10∗    −(r3(ω10∗)4−r1(ω10∗)2)sinτ2ω10∗    +(s0ω10∗−s2(ω10∗)3)cos⁡2τ2ω10∗    + s1(ω10∗)2sin2τ2ω10∗)cos⁡τ10∗ω10∗   −(n3(ω10∗)4−n1(ω10∗)2+(r0ω10∗−r2(ω10∗)3)sinτ2ω10∗     +(r3(ω10∗)4−r1(ω10∗)2)cos⁡τ2ω10∗     +(s0ω10∗−s2(ω10∗)3)sin2τ2ω10∗     − s1(ω10∗)2cos⁡2τ2ω10∗)sinτ10∗.

Obviously, if the condition (*H*_42_)  *P*_4*R*_ *Q*_4*R*_ + *P*_4*I*_ *Q*_4*I*_ ≠ 0 holds, then *Re*[*dλ*/*dτ*_1_]_*λ*=*iω*_10_*_^−1^ ≠ 0. According to the Hopf bifurcation theorem in [19], we have the following results for system (3).

**Theorem 3 .**

For system (3), if the conditions (*H*_41_)-(*H*_42_) hold and *τ*_2_ ∈ (0, *τ*_20_), then the positive equilibrium P*(S*, E*, I*, Q*, R*) of system (3) is asymptotically stable for *τ*_1_ ∈ [0, *τ*_10_*) and system (3) undergoes a Hopf bifurcation at the positive equilibrium P*(S*, E*, I*, Q*, R*) when *τ*_1_ = *τ*_10_*.

## 3. Direction and Stability of the Hopf Bifurcation

In this section, we determine the properties of the Hopf bifurcation of system (3) with respect to *τ*_1_ for *τ*_2_ ∈ (0, *τ*_20_). Throughout this section, we assume that *τ*_20_* < *τ*_10_*, where *τ*_20_* ∈ (0, *τ*_20_).

Let *τ*_1_ = *τ*_10_* + *μ*, *μ* ∈ *R* so that *μ* = 0 is the Hopf bifurcation value of system (3). Rescaling the time delay by *t* → (*t*/*τ*_1_). Let *u*_1_(*t*) = *S*(*t*) − S*, *u*_2_(*t*) = *E*(*t*) − E*, *u*_3_(*t*) = *I*(*t*) − I*, *u*_4_(*t*) = *Q*(*t*) − Q*, *u*_5_(*t*) = *R*(*t*) − R*, then system (3) can be written as a PDE in *C* = *C*([−1,0], *R* ^5^):

(46) $$\begin{array}{c} \dot{u}\left(t\right)=L_{\mu }u_{t}+F\left(\mu ,u_{t}\right), \end{array}$$

and *L*_*μ*_ : *C* → *R* ^5^, *F* : *R* × *C* → *R* ^5^ are given, respectively, by

(47) $$\begin{array}{c} L_{\mu }\phi =\left(\tau_{10}^{*}+\mu \right)\left(A^{\prime }\phi \left(0\right)+C^{\prime }\left(-\frac{\tau_{20}^{*}}{\tau_{10}^{*}}\right)+B^{\prime }\phi \left(-1\right)\right),\\ F\left(\mu ,\phi \right)=\left(\tau_{10}^{*}+\mu \right)\left(\begin{array}{c} -\beta \phi_{1}\left(0\right)\phi_{3}\left(0\right)\\ \beta \phi_{1}\left(0\right)\phi_{3}\left(0\right)\\ 0\\ 0\\ 0 \end{array}\right), \end{array}$$

with

(48) $$\begin{array}{c} A^{\prime }=\left(\begin{array}{ccccc} a_{11} & 0 & a_{13} & 0 & 0\\ a_{21} & a_{22} & a_{23} & 0 & 0\\ 0 & a_{32} & 0 & 0 & 0\\ 0 & 0 & a_{43} & a_{44} & 0\\ 0 & 0 & 0 & 0 & a_{55} \end{array}\right),\\ B^{\prime }=\left(\begin{array}{ccccc} 0 & 0 & 0 & 0 & b_{15}\\ 0 & 0 & 0 & 0 & 0\\ 0 & 0 & 0 & 0 & 0\\ 0 & 0 & 0 & 0 & 0\\ 0 & 0 & 0 & 0 & b_{55} \end{array}\right),\\ C^{\prime }=\left(\begin{array}{ccccc} 0 & 0 & 0 & 0 & 0\\ 0 & 0 & 0 & 0 & 0\\ 0 & 0 & c_{33} & 0 & 0\\ 0 & 0 & 0 & c_{44} & 0\\ 0 & 0 & c_{53} & c_{54} & 0 \end{array}\right). \end{array}$$

By the Riesz representation theorem, there exists a 5 × 5 matrix function *η*(*θ*, *μ*) : [−1,0] → *R* ^5^ whose elements are of bounded variation such that

(49) $$\begin{array}{c} L_{\mu }\phi =\overset{0}{\underset{-1}{\int }}d\eta \left(\theta ,\mu \right)\phi \left(\theta \right),\;\phi \in C. \end{array}$$

In fact, we choose

(50) η(θ,μ)={(τ10∗+μ)(A′+B′+C′),θ=0,(τ10∗+μ)(B′+C′),θ∈[−τ20∗τ10∗,0),(τ10∗+μ)B′,θ∈(−1,−τ20∗τ10∗),0,θ=−1.

For *ϕ* ∈ *C*([−1,0], *R* ^5^), we define

(51) A(μ)ϕ={dϕ(θ)dθ,−1≤θ<0,∫−10dη(θ,μ)ϕ(θ),θ=0,R(μ)ϕ={0,−1≤θ<0,F(μ,ϕ),θ=0.

Then system (46) can be transformed into the following operator equation

(52) $$\begin{array}{c} \dot{u}\left(t\right)=A\left(\mu \right)u_{t}+R\left(\mu \right)u_{t}. \end{array}$$

For *φ* ∈ *C*([−1,0], (*R* ^5^)*), we define the adjoint operator A* of *A*

(53) A∗(φ)={−dφ(s)ds,0<s≤1,∫−10dηT(s,0)φ(−s),s=0,

associated with a bilinear form

(54) 〈φ(s),ϕ(θ)〉=φ¯(0)ϕ(0) −∫θ=−10∫ξ=0θφ¯(ξ−θ)dη(θ)ϕ(ξ)dξ,

where *η*(*θ*) = *η*(*θ*, 0).

Let *ρ*(*θ*) = (1,*ρ*_2_,*ρ*_3_,*ρ*_4_,*ρ*_5_)^*T*^ *e* ^*iω*_10_*τ_10_*θ^ be the eigenvector of *A* corresponding to +*iω*_10_*τ_10_* and let ρ*(*s*) = *D*(1, *ρ*_2_*, *ρ*_3_*, *ρ*_4_*, *ρ*_5_*)*e* ^*iω*_10_*τ_10_*s^ be the eigenvector of A* corresponding to −*iω*_10_*τ_10_*. From the definition of *A*(0) and A*(0) and by a simple computation, we obtain

(55) $$\begin{array}{c} \rho_{2}=\frac{a_{21}+a_{23}\rho_{3}}{i\omega_{10}^{*}-a_{22}},\\ \rho_{3}=\frac{a_{21}a_{32}}{\left(i\omega_{10}^{*}-a_{22}\right)\left(i\omega_{10}^{*}-c_{33}e^{-i\omega_{10}^{*}\tau_{20}^{*}}\right)-a_{23}a_{32}},\\ \rho_{4}=\frac{a_{43}\rho_{3}}{i\omega_{10}^{*}-a_{44}-c_{44}e^{-i\omega_{10}^{*}\tau_{20}^{*}}},\\ \rho_{5}=\frac{i\omega_{10}^{*}-a_{11}-a_{13}\rho_{3}}{b_{15}e^{-i\omega_{10}^{*}\tau_{10}^{*}}},\\ \rho_{2}^{*}=-\frac{i\omega_{10}^{*}+a_{11}}{a_{21}},\;\;\rho_{3}^{*}=-\frac{\left(i\omega_{10}^{*}+a_{22}\right)\rho_{2}^{*}}{a_{32}},\\ \rho_{4}^{*}=-\frac{c_{54}e^{i\omega_{10}^{*}\tau_{20}^{*}}\rho_{5}^{*}}{i\omega_{10}^{*}+a_{44}+c_{44}e^{i\omega_{10}^{*}\tau_{20}^{*}}},\\ \rho_{5}^{*}=-\frac{b_{15}e^{i\omega_{10}^{*}\tau_{10}^{*}}}{i\omega_{10}^{*}+a_{55}+b_{55}e^{i\omega_{10}^{*}\tau_{10}^{*}}}. \end{array}$$

From (54), we have

(56) 〈ρ∗(s),ρ(θ)〉=D¯[1+ρ2ρ¯2∗+ρ3ρ¯3∗+ρ4ρ¯4∗+ρ5ρ¯5∗+τ10∗e−iω10∗τ10∗ρ¯5∗    +τ20∗e−iω10∗τ20∗    ×(ρ¯3∗(c33ρ3+c53ρ5)+ρ¯4∗(c44ρ4+c54ρ5))].

Let

(57) D¯=[1+ρ2ρ¯2∗+ρ3ρ¯3∗+ρ4ρ¯4∗+ρ5ρ¯5∗  +τ10∗e−iω10∗τ10∗ρ¯5∗+τ20∗e−iω10∗τ20∗  ×(ρ¯3∗(c33ρ3+c53ρ5)+ρ¯4∗(c44ρ4+c54ρ5))]−1.

Then, 〈ρ*, *ρ*〉 = 1, $\langle \rho^{*},\bar{\rho }\rangle =0$.

Next, we can obtain the coefficients determining the properties of the Hopf bifurcation by the algorithms introduced in [19] and using a computation process similar to that in [20]:

(58) g20=2βτ10∗D¯(ρ¯2∗−1)ρ(1)(0)ρ(3)(0),g11=βτ10∗D¯(ρ¯2∗−1)(ρ¯(1)(0)ρ(3)(0)+ρ(1)(0)ρ¯(3)(0)),g02=2βτ10∗D¯(ρ¯2∗−1)ρ¯(1)(0)ρ¯(3)(0),g21=2βτ10∗D¯(ρ¯2∗−1)   ×(W11(1)(0)ρ(3)(0)+12W20(1)(0)ρ¯(3)(0)     +W11(3)(0)ρ(1)(0)+12W20(3)(0)ρ¯(1)(0)),

with

(59) W20(θ)=ig20ρ(0)τ10∗ω10∗eiτ10∗ω10∗θ+ig¯02ρ¯(0)3τ10∗ω10∗e−iτ10∗ω10∗θ +E1e2iτ10∗ω10∗θ,W11(θ)=−ig11ρ(0)τ10∗ω10∗eiτ10∗ω10∗+ig¯11ρ¯(0)τ10∗ω10∗e−iτ10∗ω10∗θ+E2,

where *E*_1_ and *E*_2_ can be determined by the following equations, respectively:

(60) $$\begin{array}{c} E_{1}=2{\left(\begin{array}{ccccc} a_{11}^{\prime } & 0 & -a_{13} & 0 & -b_{15}e^{-2i\omega_{10}^{*}\tau_{10}^{*}}\\ -a_{21} & a_{22}^{\prime } & -a_{23} & 0 & 0\\ 0 & -a_{32} & a_{33}^{\prime } & 0 & 0\\ 0 & 0 & -a_{43} & a_{44}^{\prime } & 0\\ 0 & 0 & -c_{53}e^{-2i\omega_{10}^{*}\tau_{20}^{*}} & -c_{54}e^{-2i\omega_{10}^{*}\tau_{20}^{*}} & a_{55}^{\prime } \end{array}\right)}^{-1}\left(\begin{array}{c} E_{1}^{\left(1\right)}\\ E_{1}^{\left(2\right)}\\ 0\\ 0\\ 0 \end{array}\right),\\ E_{2}=-{\left(\begin{array}{ccccc} a_{11} & 0 & a_{13} & 0 & b_{15}\\ a_{21} & a_{22} & a_{23} & 0 & 0\\ 0 & a_{32} & c_{33} & 0 & 0\\ 0 & 0 & a_{43} & a_{44}+c_{44} & 0\\ 0 & 0 & c_{53} & c_{54} & a_{55}+b_{55} \end{array}\right)}^{-1}\left(\begin{array}{c} E_{2}^{\left(1\right)}\\ E_{2}^{\left(2\right)}\\ 0\\ 0\\ 0 \end{array}\right), \end{array}$$

with

(61) a11′=2iω10∗−a11,a22′=2iω10∗−a22,a33′=2iω10∗−c33e−2iω10∗τ20∗,a44′=2iω10∗−a44−c44e−2iω10∗τ20∗,a55′=2iω10∗−a55−b55e−2iω10∗τ10∗,E1(1)=−βρ(1)(0)ρ(3)(0),E1(2)=βρ(1)(0)ρ(3)(0),E2(1)=−β(ρ¯(1)(0)ρ(3)(0)+ρ(1)(0)ρ¯(3)(0)),E2(2)=β(ρ¯(1)(0)ρ(3)(0)+ρ(1)(0)ρ¯(3)(0)).

Then, we can get the following coefficients:

(62) C1(0)=i2τ10∗ω10∗(g11g20−2|g11|2−|g02|23)+g212,μ2=−Re{C1(0)}Re{λ′(τ10∗)},β2=2Re{C1(0)},T2=−Im⁡{C1(0)}+μ2Im⁡{λ′(τ10∗)}τ10∗ω10∗.

In conclusion, we have the following results.

**Theorem 4 .**

For system (3), if *μ*_2_ > 0 (*μ*_2_ < 0), the Hopf bifurcation is supercritical (subcritical). If *β*_2_ < 0 (*β*_2_ > 0) the bifurcating periodic solutions are stable (unstable). If *T*_2_ > 0 (*T*_2_ < 0), the period of the bifurcating periodic solutions increases (decreases).

## 4. Numerical Simulation

In this section, we present some numerical simulations to verify the theoretical results in Sections 2 and 3. Let *A* = 0.33, *β* = 0.75, *d* = 0.1, *η* = 0.2, *μ* = 0.3, *α* = 0.2, *γ* = 0.18, *δ* = 0.38, *ɛ* = 0.3. Then, we get the following particular case of system (3):

(63) dS(t)dt=0.33−0.75S(t)I(t)−0.1S(t)+0.2R(t−τ1),dE(t)dt=0.75S(t)I(t)−0.4E(t),dI(t)dt=0.3E(t)−0.68I(t)−0.18I(t−τ2),dQ(t)dt=0.38I(t)−0.3Q(t)−0.3Q(t−τ2),dR(t)dt=0.18I(t−τ2)+0.3Q(t−τ2)−0.1R(t),     −0.2R(t−τ1).

It is easy to verify that *R*_0_ = 2.1584 > 1. Then, we get the unique positive equilibrium P*(1.5289,0.5656,0.1973,0.1250,0.2433) of system (63). Further, we can obtain det_1_ = 2.4080 > 0, det_2_ = 3.7619 > 0, det_3_ = 2.2.0074 > 0, det_4_ = 0.1012 > 0, and det_5_ = 0.0016 > 0. That is, the condition (*H*_1_) holds.

**Figure 1:**
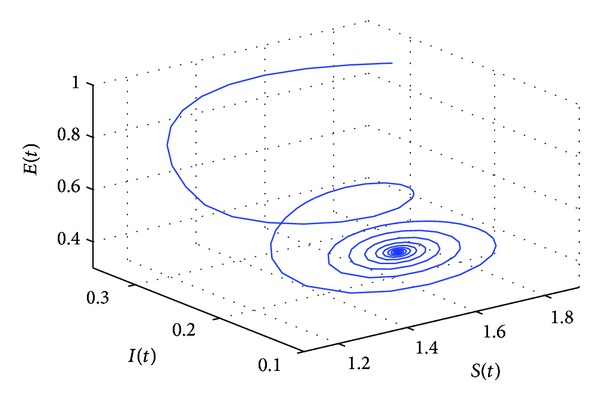
The phase plot of the states *S*, *E*, and *I* for *τ*_1_ = 7.85 < 8.4755 = *τ*_10_.

**Figure 2:**
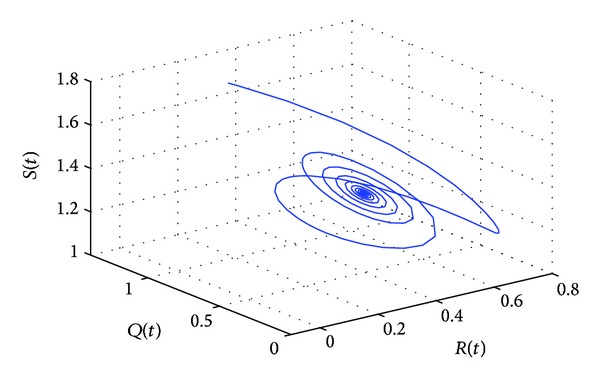
The phase plot of the states *S*, *Q*, and *R* for *τ*_1_ = 7.85 < 8.4755 = *τ*_10_.

**Figure 3:**
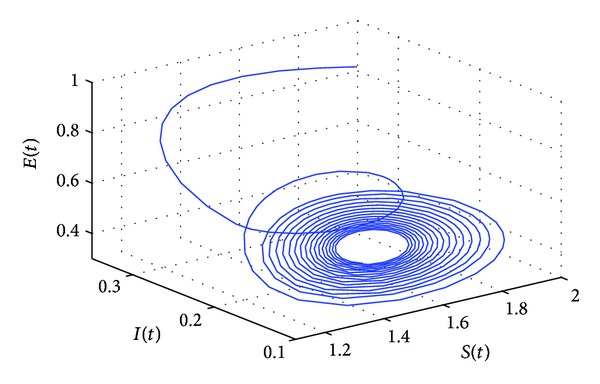
The phase plot of the states *S*, *E*, and *I* for *τ*_1_ = 9.85 > 8.4755 = *τ*_10_.

**Figure 4:**
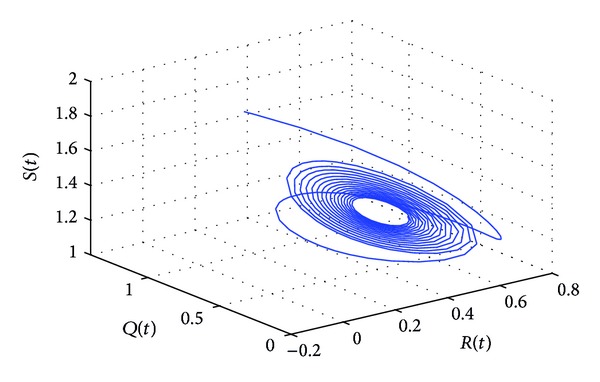
The phase plot of the states *S*, *Q*, and *R* for *τ*_1_ = 9.85 > 8.4755 = *τ*_10_.

**Figure 5:**
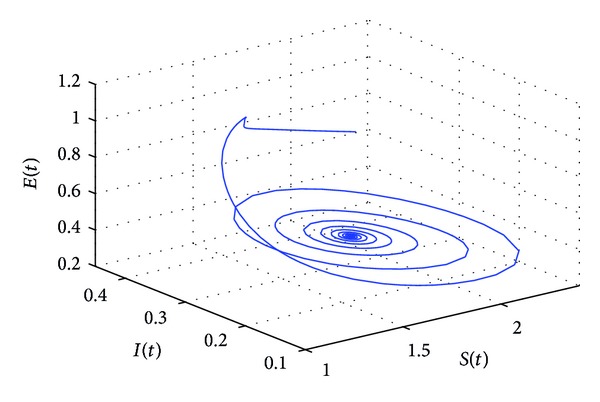
The phase plot of the states *S*, *E*, and *I* for *τ*_2_ = 7.75 < 8.1081 = *τ*_20_.

**Figure 6:**
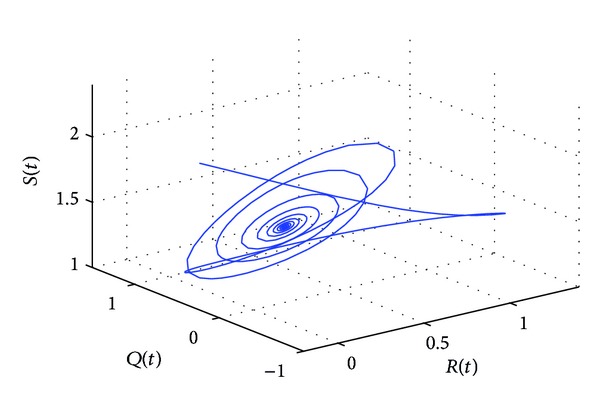
The phase plot of the states *S*, *Q*, and *R* for *τ*_2_ = 7.75 < 8.1081 = *τ*_20_.

**Figure 7:**
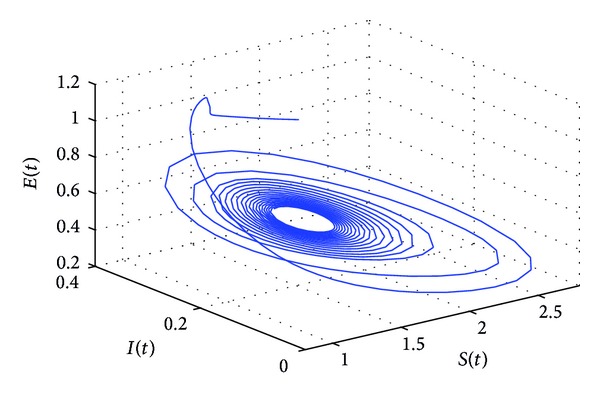
The phase plot of the states *S*, *E*, and *I* for *τ*_2_ = 9.37 > 8.1081 = *τ*_20_.

**Figure 8:**
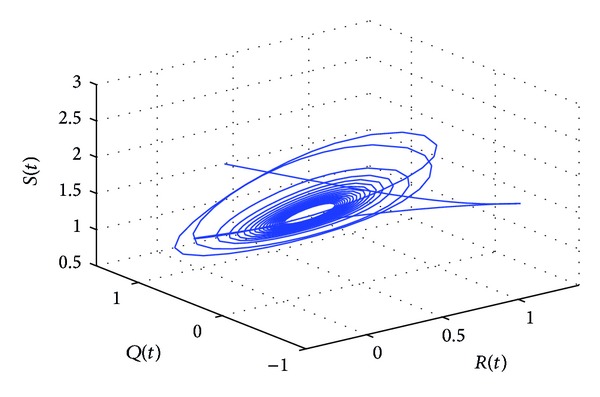
The phase plot of the states *S*, *Q*, and *R* for *τ*_2_ = 9.37 > 8.1081 = *τ*_20_.

For *τ*_1_ > 0, *τ*_2_ = 0. By some complex computation, we obtain *ω*_10_ = 1.3397, *τ*_10_ = 8.4755, and *f*_1_′(*v*_1∗_) = 2.3102 > 0. That is, the conditions (*H*_21_) and (*H*_22_) hold. According to Theorem 1, we can conclude that when *τ*_1_ ∈ [0, *τ*_10_), the positive equilibrium P*(1.5289,0.5656,0.1973,0.1250, 0.2433) of system (63) is asymptotically stable. However, when the value of *τ*_1_ passes through the critical value *τ*_10_, the positive equilibrium P*(1.5289,0.5656,0.1973,0.1250,0.2433) of system (63) will lose its stability and a Hopf bifurcation occurs at the positive equilibrium of system (63). This property can be illustrated by Figures 1–4. As can be seen from Figures 1-2, if we choose *τ*_1_ = 7.85 < *τ*_10_, it is easy to see from Figures 1-2 that the positive equilibrium P*(1.5289,0.5656,0.1973,0.1250,0.2433) of system (63) is asymptotically stable. However, if we choose *τ*_1_ = 9.85 > *τ*_10_, then the positive equilibrium P*(1.5289,0.5656,0.1973,0.1250,0.2433) loses its stability and a Hopf bifurcation occurs, which can be illustrated by Figures 3-4. Similarly, we have *ω*_20_ = 1.7690, *τ*_20_ = 8.1081 and *P*_3*R*_ *Q*_3*R*_ + *P*_3*I*_ *Q*_3*I*_ = 0.0319 > 0. Namely, the conditions (*H*_31_) and (*H*_32_) hold. The corresponding phase plots are shown in Figures 5, 6, 7, and 8.

**Figure 9:**
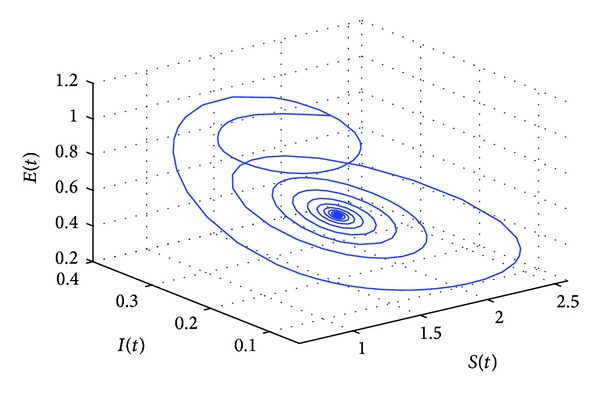
The phase plot of the states *S*, *E*, and *I* for *τ*_1_ = 5.05 < 5.6477 = *τ*_10_* and *τ*_2_ = 5.25 ∈ (0, *τ*_20_).

**Figure 10:**
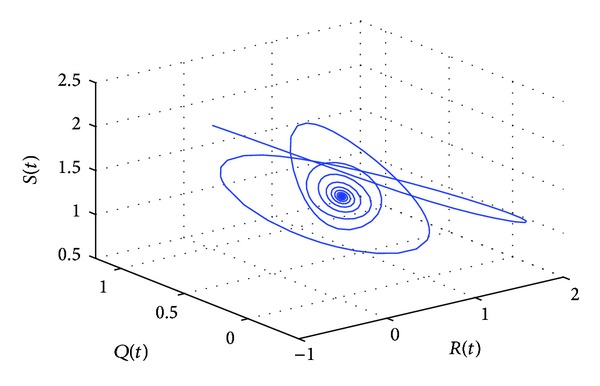
The phase plot of the states *S*, *Q*, and *R* for *τ*_1_ = 5.05 < 5.6477 = *τ*_10_* and *τ*_2_ = 5.25 ∈ (0, *τ*_20_).

**Figure 11:**
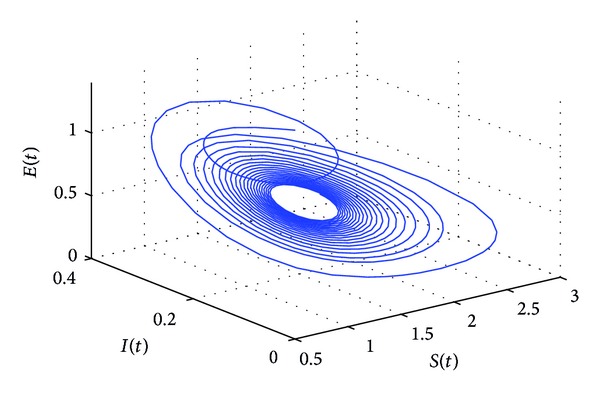
The phase plot of the states *S*, *E*, and *I* for *τ*_1_ = 6.25 > 5.6477 = *τ*_10_* and *τ*_2_ = 5.25 ∈ (0, *τ*_20_).

**Figure 12:**
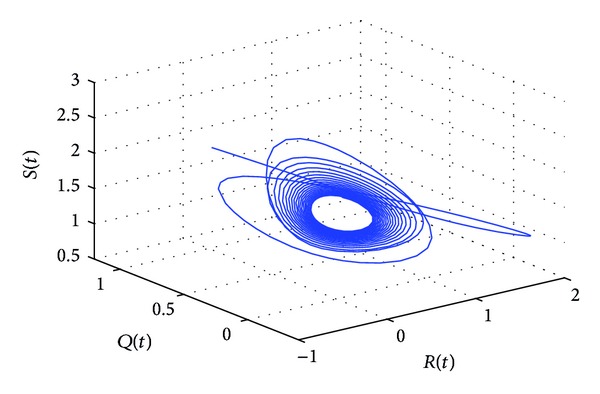
The phase plot of the states *S*, *Q*, and *R* for *τ*_1_ = 6.25 > 5.6477 = *τ*_10_* and *τ*_2_ = 5.25 ∈ (0, *τ*_20_).

For *τ*_1_ > 0, *τ*_2_ > 0 and *τ*_2_ = 5.25 ∈ (0, *τ*_20_). We obtain *ω*_10_* = 3.6529, *τ*_10_* = 5.6477 by some complex computations. The corresponding phase plots are shown in Figures 9–12. As illustrated by Figures 9-10, when *τ*_1_ = 5.05 ∈ (0, *τ*_10_*), the positive equilibrium P*(1.5289,0.5656,0.1973,0.1250,0.2433) of system (63) is asymptotically stable. However, as can be seen from Figures 11-12, the positive equilibrium P*(1.5289,0.5656,0.1973,0.1250,0.2433) of system (63) becomes unstable and a Hopf bifurcation occurs at P*(1.5289,0.5656,0.1973,0.1250,0.2433) when *τ*_1_ = 6.25 > *τ*_10_*. This property is consistent with Theorem 3. In addition, we have *λ*′(*τ*_10_*) = 0.0493 + 0.0126*i*, *C*_1_(0) = −5.8133 + 2.5756*i*. Thus, we have *μ*_2_ = 117.9168 > 0, *β*_2_ = −11.6266 < 0, *T*_2_ = −0.2711 < 0. From Theorem 4, we can conclude that the Hopf bifurcation is supercritical and the bifurcating periodic solutions are stable, and the period of the periodic solutions decreases.

## 5. Conclusions

This paper is concerned with a delayed SEIQRS model for the transmission of malicious objects in computer network. Compared with the literature [12], we consider not only the time delay due to the temporary immunity period but also the time delay due to the period that the infected computer uses to clean viruses by antivirus software. That is, the system we considered in this paper is more general than that in the literature [12]. By considering the possible combination of the two delays as a bifurcation parameter, we find that when the delay is below the corresponding critical value, the positive equilibrium of system (3) is locally asymptotically stable. However, when the delay passes through the corresponding critical value, the positive equilibrium of system (3) loses its stability and system (3) undergoes a Hopf bifurcation, which is not welcomed in networks. Furthermore, direction of the Hopf bifurcation and stability of the bifurcating periodic solutions are determined by using the normal form method and center manifold theory. Numerical simulations are presented to illustrate the theoretical analysis and results. Since the occurrence of the Hopf bifurcation is not welcomed in networks, we should control the Hopf bifurcation by some bifurcation control strategies such as the state feedback and parameter perturbation and so on. This is a further problem, which can be studied in the future.
